# A Prospective Randomized Pilot Study on the Efficacy of a Dietary Supplementation Regimen of Vitamin E and Selenium for the Prevention of Fluoroquinolone-Induced Tendinopathy

**DOI:** 10.3390/ph18040575

**Published:** 2025-04-15

**Authors:** Oana-Maria Mișcă, Liviu-Coriolan Mișcă, Bogdan Huzum, Andreea-Adriana Neamţu, Simona Cerbu, Daniel-Raul Chioibaș, Petrișor Zorin Crăiniceanu, Andrei Gheorghe Marius Motoc

**Affiliations:** 1 Plastic and Reconstructive Surgery Department—Casa Austria, Pius Brînzeu Clinical County Emergency Hospital Timişoara, Liviu Rebreanu Boulevard, No. 156, 300723 Timișoara, Romania; oamarginean@gmail.com (O.-M.M.); zcrainiceanu@gmail.com (P.Z.C.); 2 Doctoral School Department, “Victor Babeș” University of Medicine and Pharmacy of Timișoara, Eftimie Murgu Square, No. 2, 300041 Timişoara, Romania; liviu.misca@umft.ro; 3 Trauma & Orthopaedics Department, Cork University Hospital, Wilton, T12 DC4A Cork, Ireland; 4 Department of Orthopedics and Traumatology, Faculty of Medicine, “Grigore T. Popa” University of Medicine and Pharmacy, Universității Str., No. 16, 400347 Iasi, Romania; 5 Department of Pathology, Pius Brînzeu Clinical County Emergency Hospital Timişoara, Liviu Rebreanu Boulevard, No. 156, 300723 Timișoara, Romania; 6 Department of Toxicology, “Victor Babeș” University of Medicine and Pharmacy of Timișoara, Eftimie Murgu Square, No. 2, 300041 Timișoara, Romania; 7 Research Centre for Pharmaco-Toxicological Evaluation, “Victor Babeș” University of Medicine and Pharmacy of Timișoara, Eftimie Murgu Square, No. 2, 300041 Timișoara, Romania; 8 Department of Radiology and Medical Imaging, “Victor Babeș” University of Medicine and Pharmacy of Timișoara, Eftimie Murgu Square, No. 2, 300041 Timisoara, Romania; cerbusimona@yahoo.com; 9 Department IX of Surgery, “Victor Babeș” University of Medicine and Pharmacy of Timișoara, Eftimie Murgu Square, No. 2, 300041 Timișoara, Romania; chioibas.raul@umft.ro; 10 Department X of General Surgery, “Victor Babeș” University of Medicine and Pharmacy of Timișoara, Eftimie Murgu Square, No. 2, 300041 Timișoara, Romania; 11 Department of Anatomy and Embryology, “Victor Babeș” University of Medicine and Pharmacy of Timișoara, Eftimie Murgu Square, No. 2, 300041 Timișoara, Romania; amotoc@umft.ro

**Keywords:** tendinopathy, fluoroquinolone, levofloxacin, vitamin E, selenium, Achilles tendon thickness, VAS score, VASA-A score

## Abstract

**Background**: Fluoroquinolone-induced tendinopathy is a clinically significant adverse effect associated with this class of antibiotics, particularly affecting the Achilles tendon. Despite its growing recognition, the precise pathophysiological mechanisms remain incompletely understood, with hypotheses referencing increased matrix metalloproteinase activity, collagen degradation, and oxidative stress. **Methods**: This prospective randomized pilot study evaluates the potential protective effectiveness of vitamin E and selenium supplementation in mitigating fluoroquinolone-induced tendinopathy. The study was conducted on 25 patients receiving 500 mg/day levofloxacin antibiotherapy, randomly divided into a control group and an experimental group—vitamin E (400 IU/day) and selenium (200 µg/day), oral supplementation for 28 days. Clinical assessment of the pain level through the VAS score and of functionality through the VISA-A score was performed, alongside ultrasound imaging of the Achilles tendon. To assess potential toxicity and ensure adherence to the supplementation protocol, serial biochemical analyses of serum vitamin E and selenium were performed at predetermined intervals. **Results**: A significant improvement was observed in pain scores (*p* = 0.0120) and functional outcomes (*p* = 0.0340) when comparing the control and experimental groups at the three-month follow-up. Ultrasound analysis revealed reduced tendon thickness and neovascularization, supporting structural recovery. Although the incidence of tendinopathy was lower in the interventional group (13.3% vs. 40%), statistical significance was not reached, possibly due to the small sample size. **Conclusions**: These findings suggest that antioxidant supplementation with vitamin E and selenium may provide a protective effect against fluoroquinolone-induced tendinopathy, warranting further investigation in larger randomized clinical trials.

## 1. Introduction

Tendinopathies encompass a range of alterations that occur in injured tendons, which lead to pain and impaired function, marked by changes in the microstructure, composition, and cellularity of the tendon [1]. Physiologically, a tendon presents a network of tightly and parallelly arranged collagen fibers and a few cells, primarily tenocytes, aligned along the fibers’ length [2]. Pathologically, a tendon affected by tendinopathy displays fragmented collagen fibers, disordered collagen bundles, an accumulation of glycosaminoglycans, and an increased microvascular compartment accompanied by neoinnervation [3,4]. These changes negatively impact the material properties, impacting patients’ ability to endure, store, and transmit significant force necessary for daily activities through the impaired structure [1]. Tendinopathy often arises from overuse of the anatomical structure, triggering a series of pathological processes that result in pain, either localized or diffuse swelling, compromised tissue integrity, and reduced performance [5].

The incidence of tendinopathy in the lower limb (e.g., Achilles, patellar, and plantar heel pain) is reported to be around 1–2% in the general adult population (18–65 years old age group) throughout the entire lifetime [1]. In addition to sports injuries, the multifactorial elements implicated in the development of tendinopathies include associated pathologies such as obesity, hypercholesterolemia, and diabetes mellitus, which have been shown to influence the incidence and severity of tendinopathic lesions, as well as the recovery prognosis of this pathology [6,7,8]. Furthermore, the use of certain antibiotics, such as fluoroquinolones, is associated with an increased risk of tendinopathy and tendon rupture, with a variable excess risk when compared to individuals not undergoing antibiotic therapy with this class of medications [9,10,11,12].

Fluoroquinolones are widely used antibiotics due to their excellent antibiotic penetration and prolonged half-life [13]. They have been successfully employed in both Gram-positive and Gram-negative bacterial infections, with antibiograms demonstrating increased sensitivity of numerous pathogens. The first reported case of tendinopathy (particularly of the Achilles tendon) associated with fluoroquinolone treatment was described in 1983 [14,15]. Currently, it is conceived as a relatively common complication encountered in clinical practice and relatively challenging to treat [16,17,18].

The underlying pathophysiological mechanism of fluoroquinolone-induced tendinopathy is not currently fully elucidated, although several concepts are suggested. One of the hypotheses refers to the increased expression of matrix metalloproteinases (MMP), which induces collagen degradation [10,19]. Some studies describe hyaline and mucoid degeneration leading to chondroid metaplasia of tenocytes and alterations in mucopolysaccharide and collagen fibers [20]. Others mention reduced vascular caliber in the paratenon, resulting in ischemic lesions [21,22,23]. Alternatively, it is suggested that as bacterial type II topoisomerases are targeted by fluoroquinolones, mitochondrial dysfunction implicated in some adverse events may be explained by the interaction between fluoroquinolones and human topoisomerase IIα or IIβ, disrupting mitochondrial DNA replication [24].

To avoid fluoroquinolone-induced tendinopathies, studies suggest a correlation between fibroblast cytotoxicity and oxidative stress. Thus, concomitant administration of vitamin E and selenium with the antibiotic treatment may offer potential benefits, employing antioxidant effect as a protective strategy [25,26].

Vitamin E (α-tocopherol) and selenium are well-known antioxidants demonstrated efficacy in mitigating oxidative damage and preserving tissue integrity. Vitamin E functions as a liposoluble antioxidant that protects cellular membranes from oxidative injury [27], while selenium, as an essential component of selenoproteins, contributes to enzymatic antioxidant defense mechanisms [28]. The efficacy of vitamin E and selenium supplementation has been attributed to their ability to reduce reactive oxygen species (ROS) levels and attenuate apoptotic activity [29,30]. Nevertheless, clinical evidence is currently insufficient to certify this hypothesis [25,26,31,32].

Considering the synergistic antioxidant effect of vitamin E and selenium, the original research presented in this article aimed to evaluate the protective effects of a supplementation regimen in mitigating fluoroquinolone-induced tendinopathy. By employing a prospective design, we sought to assess the impact of these antioxidants through assessment of clinical symptoms—localized pain reported through the visual assessment scale (VAS), functional outcomes—reported through the Victorian Institute of Sport Assessment-Achilles scale (VISA-A), and tendon integrity—analyzed through ultrasonography. The results are meant to contribute to the development of adjuvant strategies employed in the reduction of fluoroquinolone-associated musculoskeletal adverse effects, hoping to improve patient outcomes.

## 2. Results

### 2.1. Patient Characteristics

Patients included in the study (Table 1; raw data provided in the Supplementary Materials section Table S1) were 46.24 ± 10.69 years old (8 males and 17 females), with control Group C being 44.80 ± 11.73 years old (4 males and 6 females) and experimental Group ES being 47.20 ± 10.26 years old (4 males and 11 females). There was no statistically significant difference (*p* = 0.6050) at the level of significance α = 0.05, employing the unpaired *t*-test with Welch’s correction (assumes Gaussian distribution, does not assume equal standard deviation), between the control and experimental group.

### 2.2. Serum Vitamin E and Selenium Levels

**Figure 1:**
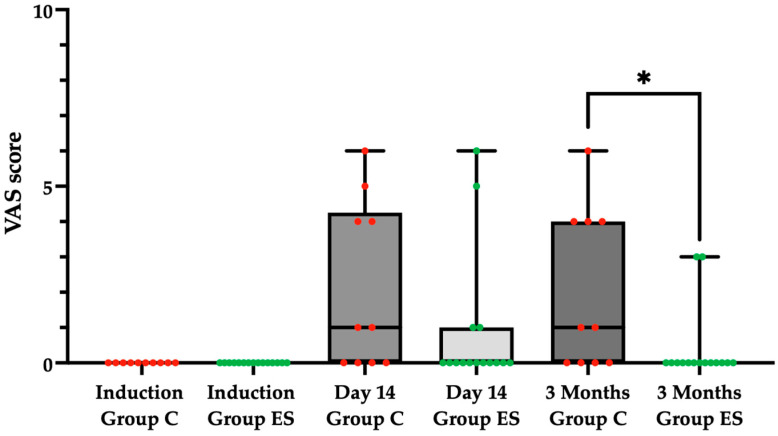
VAS score for pain in the control Group C (red) and the experimental Group ES (green) at induction, at 14 days, and at 3 months. Statistically significant difference (*p* = 0.0120) at the level of significance α = 0.05, employing the Mann–Whitney test, denoted as *.

Serum vitamin E and selenium levels did not indicate abnormal elevations due to the 28-day supplementation regimen adopted by the experimental Group ES—selenium 200 µg/day and vitamin E 400 IU/day. Normal serum values are 5.00–20.00 mg/L. Selenium levels remained within the reference range, while vitamin E showed a slight increase above the reference values without associated clinical symptoms (Table 2). Nevertheless, there was a statistically significant difference (*p* < 0.0001) at the level of significance α = 0.05, employing the paired *t*-test (assumes Gaussian distribution), between the vitamin E and the selenium level at induction and at the end of the 28-day cure, indicating a consistent difference between paired values (Figure 1).

### 2.3. Clinical Evaluation—Symptoms

All patients were assessed using the VAS and VISA-A scales at induction, at 14 days, and at 3 months post-treatment. At induction, no patient exhibited clinically significant levels of pain in the Achilles tendon.

At the second evaluation (14 days), in the control Group C the average VAS score obtained was 2.10 ± 2.38, while in the experimental Group ES the average score obtained was 0.87 ± 1.92, without a statistically significant difference. At the third evaluation (3 months), in the control Group C the average VAS score obtained was 2.00 ± 2.26, while in the experimental Group ES the average score obtained was 0.40 ± 1.06. At the 3 month evaluation, there was a statistically significant difference (*p* = 0.0120) at the level of significance α = 0.05 between the control Group C and experimental Group ES in regards to the VAS score, employing the Mann–Whitney test, (Figure 1).

**Figure 2:**
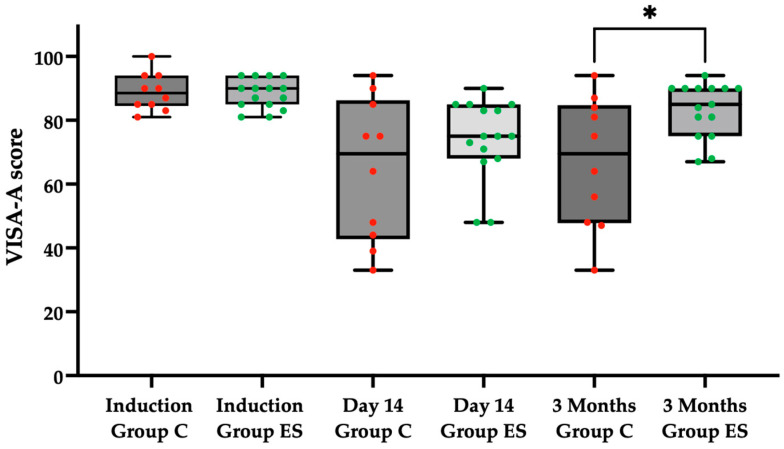
VISA-A score for pain in the control Group C (red) and the experimental Group ES (green) at induction, at 14 days, and at 3 months. Statistically significant difference (*p* = 0.0340) at the level of significance α = 0.05, employing the Mann–Whitney test, denoted as *.

At the initial evaluation (baseline), in the control Group C the average VISA-A score obtained was 88.9 ± 5.86, while in the experimental Group ES the average score obtained was 88.33 ± 4.64, without a statistically significant difference. At the second evaluation (14 days), in the control Group C the average VISA-A score obtained was 64.7 ± 22.35, while in the experimental Group ES the average score obtained was 74.07 ± 12.62, without a statistically significant difference. At the third evaluation (3 months), in the control Group C the average VISA-A score obtained was 66.9 ± 20.34, while in the experimental Group ES the average score obtained was 83.33 ± 8.60. At the 3 month evaluation, there was a statistically significant difference (*p* = 0.0340) at the level of significance α = 0.05 in regards to the VISA-A score, employing the Mann–Whitney test, between the control Group C and experimental Group ES (Figure 2).

### 2.4. Paraclinical Evaluation—Ultrasound

**Figure 3:**
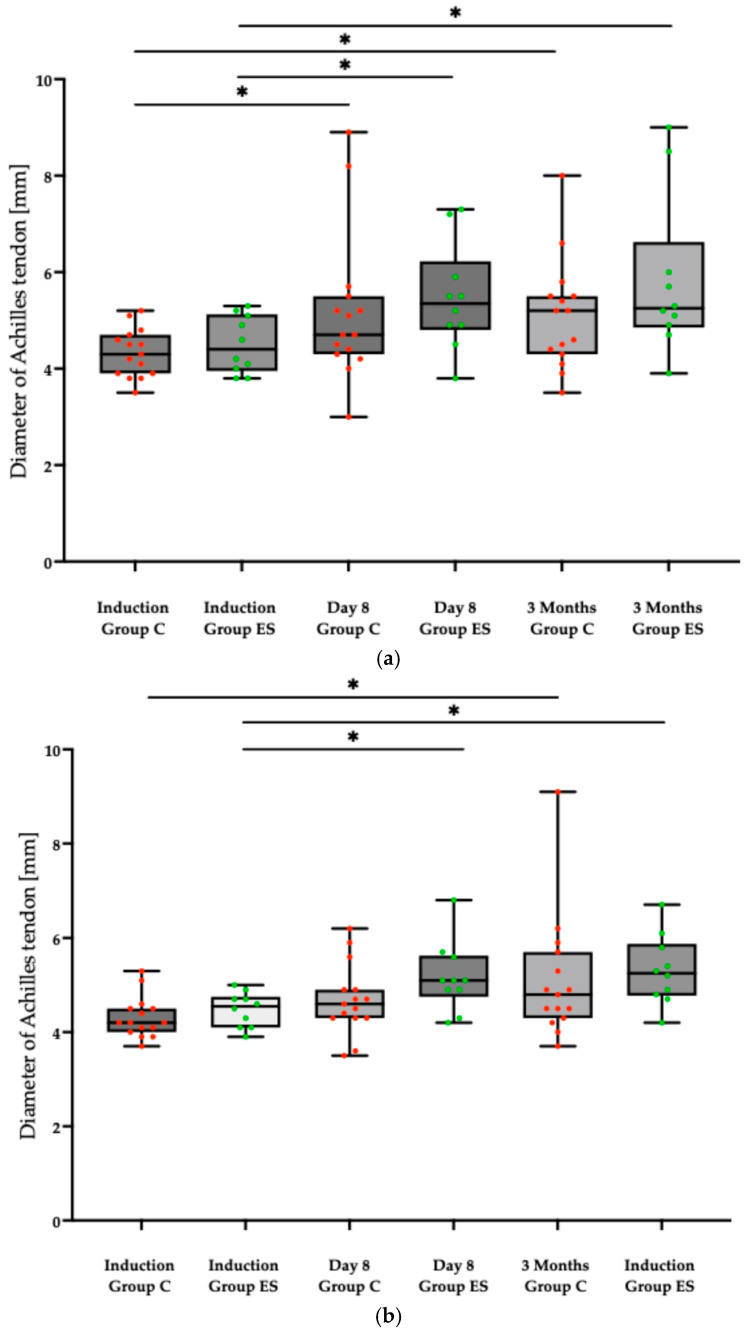
Longitudinal incidence ultrasound measurements of the diameter [mm] of the Achilles tendon in the control Group C (red) and the experimental Group ES (green) at induction, day 8, and 3 months, for both the left (**a**) and the right (**b**) leg. Statistically significant differences (*p* < 0.05) at the level of significance α = 0.05, employing the paired *t*-test (assumes Gaussian distribution), denoted as *.

**Figure 4:**
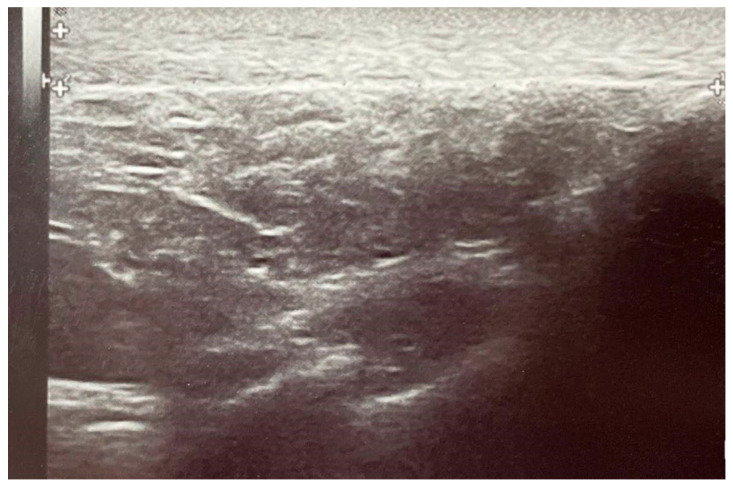
Longitudinal incidence ultrasound at treatment induction showing the physiological appearance of the Achilles tendon—diameter of 4.8 mm.

**Figure 5:**
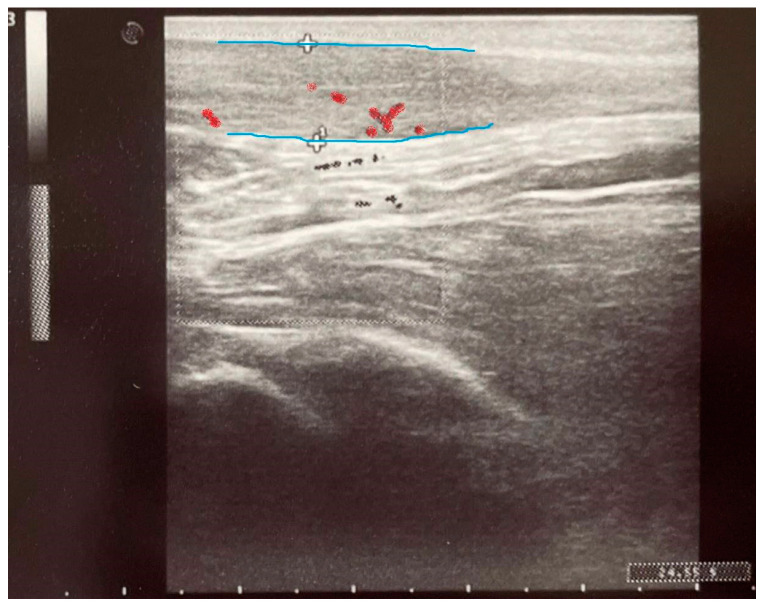
Longitudinal incidence ultrasound and color Doppler at 8 days showing fusiform thickening (blue lines) of the Achilles tendon with neovascularization (red)—diameter of 9.1 mm.

**Figure 6:**
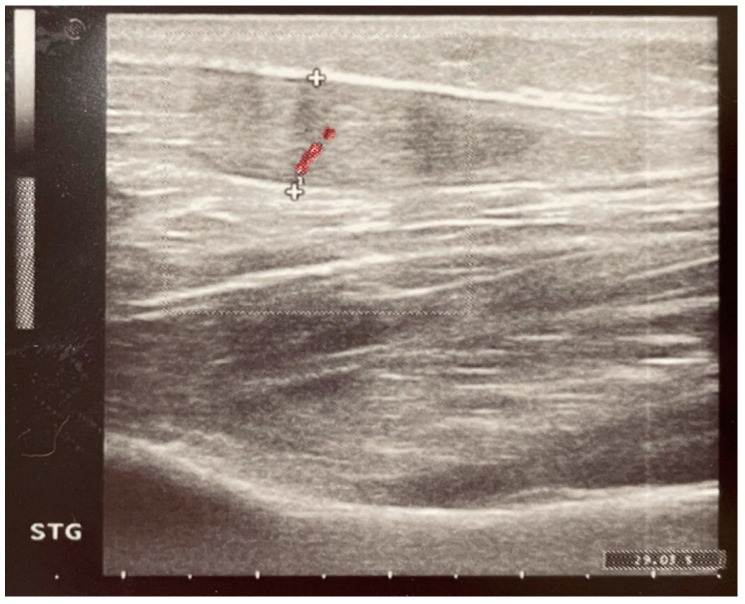
Longitudinal incidence ultrasound and color Doppler at 3 months showing an improvement in Achilles tendon thickness with decreased vascularization (red)—diameter of 6.6 mm.

Baseline ultrasound imaging at induction showed no evidence of tendinopathy prior to fluoroquinolone administration (Figure 3 and Figure 4). However, ultrasound examinations at day 8 using Doppler imaging revealed localized fusiform thickening of the Achilles tendon in six patients compared to baseline, associated with fibrillar ruptures and neovascularization (Figure 5). At the 3-month follow-up, structural improvements in the Achilles tendon were observed in the two patients treated with vitamin E and selenium, characterized by reduced tendon diameter and enhanced structural integrity (Figure 6). Various statistically significant differences (*p* < 0.05) between evaluations were observed at the level of significance α = 0.05, employing the paired *t*-test (assumes Gaussian distribution) (Figure 3, Table 3).

### 2.5. Diagnosed Tendinopathy

In the entire study group, six patients developed tendinopathy:

- Group C (control): four patients (40.0%)
- Group ES (experimental): two patients (13.3%).

This difference is not statistically significant (*p* = 0.1262) at the level of significance α = 0.05, employing the chi-square test. This is due to the low number of participants included in this pilot prospective randomized cohort study.

### 2.6. Study Model Validation

In the study group, the VAS and VISA-A scores are correlated with the tendon thickness measured through ultrasonography (Table 4). The statistically significant correlation (*p* < 0.05) at the level of significance α = 0.05 was observed when employing the nonparametric Spearman test (does not assume Gaussian distribution) and is depicted in Table 4.

## 3. Discussion

Clinically, fluoroquinolones represent a valuable resource due to their wide spectrum of activity, bioavailability, extensive tissue penetration, and successful antibacterial outcomes [33]. Nevertheless, the toxicity of this antibiotic class is currently well documented and should be considered and mitigated whenever possible. Mild adverse reactions such as diarrhea, nausea, and headaches have been recognized as common across all fluoroquinolones since their initial approval by the Food and Drug Administration [34]; however, more serious concerns have been identified over time [35]. Among the serious side effects, toxicity to the cardiovascular, nervous, musculoskeletal, renal, and hepatic systems has been observed [35], rendering the usage of this class as a last-resort antibiotic [36].

Fluoroquinolone-induced Achilles tendinopathy was first reported in 1983 by Dr. R Bailey from New Zealand [15] and thereafter described in numerous clinical studies [10,11,12]. Dose-independent fluoroquinolone-induced tendinopathies were described [37], mainly upon usage of ciprofloxacin, gemifloxacin, levofloxacin, moxifloxacin, norfloxacin, and ofloxacin [38], mainly (about 95%) affecting the Achilles tendon [39]. Taking this into account, the current prospective randomized pilot cohort study focused on the Achilles tendon is in alignment with the scientific literature. Moreover, among fluoroquinolones, levofloxacin exhibits the highest risk of tendinopathy [40], demonstrating the potential of the study design. Research suggests that matrix metalloproteinases 1 and 2 (MMP1 and MMP2) are activated by fluoroquinolones [40]. Subsequently, these metalloproteinases degrade type I collagen, reducing the overall size and number of collagen fibrils, thus weakening the tendon [23]. Alternatively, underexpression of integrins in tenocytes could destabilize the extracellular matrix (ECM) [40,41]. Moreover, tenocyte apoptosis could be caused by the increased concentration of reactive oxygen species (ROS), either due to fluoroquinolone-induced mitochondrial dysfunction [42] or due to ROS production in the targeted bacteria, where the drugs activate genes contributing to oxidative stress [43].

Fluoroquinolone-induced tendinopathies are exhibited by patients with an average age of about 64 years old, with a 2:1 male-to-female ratio [44], two decades older than the patients included in our pilot study, where the average age was 46.24 ± 10.69 years old, and following a reversed ratio of 1:2 male-to-female due to the requirements for fluoroquinolone antibiosis in the study period in the hospital. Despite the general prevalence of fluoroquinolone-induced tendon injuries being low (0.14–0.4%), their risk of development is significantly increased in patients with chronic renal failure, hemodialysis, and systemic corticosteroid therapy [45]. In the current pilot study, a high incidence of fluoroquinolone-induced tendinopathy was observed, with six cases diagnosed symptomatically and through ultrasonography among the 25 patients enrolled, with 40.0% of the patients in the control group and 13.3% in the experimental group. Nevertheless, no cases of spontaneous Achilles tendon ruptures were identified among the patients included in this study.

The choice of using the VAS score for pain in the symptomatic assessment of tendinopathy is common in medical practice and scientific literature [46,47,48,49] due to its simplicity and sensitivity to changes in pain perception, minimal patient instruction requirements, and quickness of administration, all of which are crucial for monitoring the progression of pain [50]. The VISA-A questionnaire is a specialized tool designed to assess the severity of Achilles tendinopathy and is particularly valuable in both clinical and research settings [51]. The VISA-A is structured around eight questions that focus on pain, function in daily activities, and the ability to participate in sports, and it has been validated for different populations, including non-surgical patients, pre-surgical patients, and control groups [52,53,54]. Therefore, our study employed both of these well-established scores for symptomatic and functional assessment, aligning with the current trends in clinical setting and research. Nevertheless, currently, the 13-item TENDINopathy Severity assessment–Achilles (TENDINS-A) is gaining traction in the field due to its superiority [55]. However, it was not included in the current assessment due to its development after this study was conducted. In our study, the VAS and VISA-A scores correlated with the tendon thickness measured through ultrasonography, as an average of the left and right legs (Table 4), therefore validating the outcome.

The results of this study suggest that vitamin E and selenium supplementation may provide a protective effect against fluoroquinolone-induced tendinopathy. This is supported by the scientific literature, where the underlying biochemical mechanisms are hypothesized to act through the mitigation of oxidative stress leading to improvement in tendon structural integrity. Compared to the control group, patients receiving antioxidant supplementation exhibited significantly lower pain scores (VAS, *p* = 0.0120) and improved functional recovery (VISA-A, *p* = 0.0340) at the three-month follow-up. Additionally, ultrasound imaging revealed a reduction in tendon thickness and neovascularization, further supporting a beneficial effect on tendon remodeling (Figure 4, Figure 5 and Figure 6). The study demonstrates a statistically significant strong correlation between ultrasound measurements of tendon thickness and the severity of symptomatic pain, supporting the diagnosis of tendinopathy following levofloxacin administration (Table 4). The adverse effects showed a decrease at the three-month follow-up in the experimental Group ES, whereas the control Group C exhibited a relatively stable progression with no marked improvement. Both ultrasound imaging and clinical assessments confirm the progressive onset of tendinopathy in the group that received levofloxacin alone, in contrast to the interventional group where improvements were noted.

To our knowledge, specific research directly linking selenium and vitamin E supplementation to the prevention or reduction of fluoroquinolone-induced tendinopathy is not conclusively established in the available literature. Nevertheless, the observed improvement in tendon structure in the supplemented group supports the hypothesis that antioxidant therapy may counteract ECM degradation and tenocyte apoptosis by reducing ROS accumulation [56,57]. One study inferred that ciprofloxacin-induced fibroblast apoptosis and mitochondrial dysfunction could be partially reversed by vitamin E administration [58]. Theoretically, the synergistic effect of selenium and vitamin E observed in other contexts could aid in tendinopathy prevention by following the well-established antioxidant mechanisms. Selenium is an essential trace mineral and acts as an essential cofactor for glutathione peroxidase, selenoprotein P, and thioredoxin reductase, all involved in free radical scavenging and redox balance maintenance [59]. Vitamin E is a liposoluble essential micronutrient that interacts with the lipids to avoid the formation of hydroperoxides [60].

The dosage of vitamin E and selenium used in this study are meant for a short-term intervention (28 days), designed to deliver maximum benefits for the patients from the first day of fluoroquinolone administration. While the recommended daily allowance (RDA) was exceeded in this study, the dosage lays well below the tolerable upper intake levels (UL). For vitamin E, the UL is 1000 mg/day or 1500 UI/day [61]. Low doses of 30 mg/day were observed to have little to no effect on plasma and tissue α-tocopherol concentrations, which changed in the case using 300 mg/day [62]. In this context, combined with the wide availability of vitamin E supplements with a 400 UI concentration in Romania, this dosage was selected. For selenium, the UL was set at 400 μg/day, seven times higher than the RDA of 55 μg/day [61,63]. Nevertheless, intake of selenium above the normal nutritional range (e.g., 200 μg/day) has been deemed to confer additional health benefits [64]; thus, supplements in this dosage are readily available.

Our prospective randomized pilot study provides clinical evidence for the potential of vitamin E and selenium supplementation to reduce pain, improve function, and enhance tendon integrity in patients undergoing fluoroquinolone therapy, aiming to minimize fluoroquinolone-induced tendinopathy. To our knowledge, this represents the first human-based interventional study utilizing this particular methodological approach. The results support vitamin E and selenium synergistic coupling as antioxidant complementary therapy as potential intervention against fluoroquinolone-induced tendinopathy. Nevertheless, the authors would like to acknowledge the reduced sample size and relatively short follow-up period as limitations of the presented data, leading to reduced statistical power of the study, with increased type I and type II errors. Moreover, potential misclassification and selection bias could render the results less reliable. In order to establish vitamin E and selenium antioxidant regimens as a routine complementary therapy to fluoroquinolone treatment, larger blind multicentric randomized clinical trials should be conducted with a longer follow-up.

## 4. Materials and Methods

A prospective randomized pilot study was conducted for 9 months, in the period 1 February and 31 October 2021, at the Clinical County Emergency Hospital “Pius Brînzeu” Timișoara.

Inclusion criteria:

- Patients of the Emergency Clinical Hospital “Pius Brînzeu” Timișoara, in the period 1 February and 31 October 2021.
- Patients aged between 30 and 60 years old.
- Patients presenting with microbial infections that required initiation of antibiotic therapy according to the antibiogram, with a minimum dose of 500 mg/day of levofloxacin for 7 consecutive days.
- Patients consenting to take part in the study.

Exclusion criteria:

- Patients with predisposing risk factors, such as corticosteroid therapy, renal insufficiency, diabetes mellitus, or a history of organ transplantation, were excluded to minimize confounding variables.
- Patients who had previously undergone Achilles tendon surgery were excluded.
- Patients exhibiting pain at the level of the Achilles tendon prior to fluoroquinolone administration were excluded.

A total of 25 patients were enrolled in the study and divided as follows:

- Control group (Group C)—10 patients. The patients received standard levofloxacin treatment without antioxidant supplementation (placebo pills were not provided).
- Experimental group (Group ES)—15 patients. The patients were administered vitamin E (400 IU/day) and selenium (200 µg/day), orally, for a duration of 28 days, starting on the first day of levofloxacin prescription (patients chose their preferred supplements with the recommended dosage from the pharmacy).

For the randomization, 25 sealed envelopes containing the Group C (N = 10) and Group ES (N = 15) information were mixed prior to the beginning of the study. Upon finding a patient that fit the inclusion and exclusion criteria, a random envelope was picked by the principal investigator, assigning the patient to the study group. No party conducting or taking part in this pilot study was blinded.

All participants were hospitalized at the Emergency Clinical Hospital “Pius Brînzeu” Timișoara and received antibiotic therapy with levofloxacin 500 mg/day for 7 days.

The study protocol employed periodic evaluation, both clinical assessment of pain (1) and sonographic assessment of Achilles tendon thickness (2), and the vitamin E and selenium serum levels were monitored (3). The framework for evaluations included 5 consults, distributed temporally as follows:

1. Baseline (prior to treatment initiation)—(1), (2), (3);
2. Day 8 (following 7 days of levofloxacin therapeutic regimen)—(2);
3. Day 14 (mid-treatment evaluation)—(1);
4. Day 28 (end of vitamin E and selenium supplementation)—(3);
5. Three months post-treatment assessment—(1), (2).

The clinical assessments utilized validated scoring systems applied for pain level and functionality of Achilles tendinopathy: the Visual Analog Scale (VAS) [65] and the VISA-A [66,67].

The ultrasound examination utilized an MyLab (Esaote, Noida, Uttar Pradesh, India) ultrasound machine with a linear probe (4–13 MHz) and a General Electric Venue 50 ultrasound machine with a linear probe (6–15 MHz), both equipped with grayscale and color Doppler. The evaluation was conducted by two independent evaluators, maintaining consistent landmarks for measurements. Patients were examined in 90° dorsiflexion, with their feet positioned at the distal end of the examination table, and measurements were taken at a distance of 45 mm from the calcaneal tendon insertion. To highlight pathological neovascularization and avoid false-positive results of vascularity using color Doppler, measurements were taken after 30 min of sustained physical exertion [68].

All participants were informed about the study protocol and provided informed consent before study initiation. The study was conducted in accordance with the Declaration of Helsinki with respect to the rules of good clinical practice in biomedical research, and the protocol was approved by the Ethics Committee of the University of Medicine and Pharmacy “Victor Babeș” Timișoara, Romania (Approval No.67/17.12.2020) and by the Institutional Review Board and Ethics Committee for Scientific Research of the Emergency Clinical County Hospital “Pius Brînzeu” Timișoara, Romania (Approval No. 223/05.02.2021).

The current prospective randomized pilot study is reported in accordance with the CONSORT 2010 guidelines; check-list provided in the Supplementary Materials section Table S2.

The statistical analysis was performed using the software GraphPad Prism Version 10.2.3 (347), 21 April 2024 of Prism 10 for macOS (USA). Tests were chosen based on the specific type of data and the assumptions underlying their distributions, ensuring that the statistical analysis is both appropriate and robust for interpretation:

- Unpaired *t*-test with Welch’s correction: to compare the mean ages between the control and experimental groups. The choice of Welch’s correction suggests that the test does not assume equal variances between the two groups, making it suitable for data where the standard deviations might differ.
- Paired *t*-test: to analyze the serum vitamin E and selenium levels within the experimental group before and after the supplementation regimen. This test assumes that the data are normally distributed and compares the means of two related groups to determine if there is a statistically significant difference between them.
- Mann–Whitney nonparametric test: to compare the VAS and VISA-A scores between the control and experimental groups at different time points. The choice of a nonparametric test indicates that the data may not follow a normal distribution, making the Mann–Whitney test a robust option for comparing medians.
- Chi-square test: to assess the significance of the difference in the incidence of tendinopathy between the control and experimental groups. This test is appropriate for categorical data, providing a method to determine if the distribution of cases across the categories is due to chance.
- Spearman correlation test: to investigate the relationship between tendon thickness and VAS/VISA-A scores. Being a nonparametric measure of rank correlation, it assesses how well the relationship between two variables can be described using a monotonic function, suitable for data that do not necessarily follow a normal distribution.

## 5. Conclusions

Fluoroquinolones, while clinically valuable for their broad antibacterial efficacy and pharmacokinetic properties, are associated with significant risks of systemic toxicity affecting multiple organ systems, most notably the musculoskeletal system. Notably, fluoroquinolone-induced Achilles tendinopathy represents a serious adverse effect, with mechanisms likely linked to changes in matrix metalloproteinase activities and tenocyte function, potentially exacerbated by oxidative stress. This pilot prospective randomized study explored the protective potential of vitamin E and selenium supplementation against fluoroquinolone-induced tendinopathy. Preliminary findings suggest that antioxidant supplementation might mitigate some of the deleterious effects on the Achilles tendon by improving structural integrity and reducing symptoms, as evidenced by lower pain scores and better functional outcomes in the supplemented group compared to controls. While promising, these results must be interpreted with caution due to the pilot nature of the study and its limitations, including a small sample size and short follow-up period. Further research is essential to substantiate these findings and potentially incorporate antioxidant therapy as a routine prophylactic measure alongside fluoroquinolone usage. Such studies could provide crucial insights into the mechanistic pathways and therapeutic strategies to prevent or reduce the severity of fluoroquinolone-induced tendinopathies, ultimately enhancing patient outcomes in clinical settings.

## Figures and Tables

**Table 1:** Patient characteristics.

Characteristic	Control Group C	Experimental Group ES
Number of patients	10	15
Number of males/females	4/6	4/11
Patients from rural/urban area	3/7	2/13
Age	44.80 ± 11.73 years	47.20 ± 10.26 years
Achilles tendon thickness	4.32 ± 0.46 mm	4.49 ± 0.48 mm

Note: Values reported as mean ± standard deviation (SD).

**Table 2:** Average values of serum selenium and vitamin E concentrations in the experimental Group ES (total number of patients N = 15).

	Serum Concentration at Induction (Baseline)	Serum Concentration at 28 Days of Supplementation	Reference Values of the Laboratory for General Population
Vitamin E (****)	14.57 ± 3.83 mg/L 14.57 ± 0.99 mg/L	23.53 ± 3.38 mg/L 23.53 ± 0.87 mg/L	5.00–20.00 mg/L
Selenium (****)	95.90 ± 9.80 µg/L 95.90 ± 2.53 µg/L	115.86 ± 7.57 µg/L 115.86 ± 1.96 µg/L	23.00–190.00 µg/L

Note: Serum concentrations reported as mean ± standard deviation (SD), followed by mean ± standard error. Statistically significant difference in between the induction and the 28th day (*p* < 0.0001) at the level of significance α = 0.05, employing the paired *t*-test (assumes Gaussian distribution), denoted as ****.

**Table 3:** Achilles tendon thickness in the control Group C (total number of patients N = 10) and the experimental Group ES (total number of patients N = 15), recorded for both the left and the right legs.

	Achilles Tendon Thickness (Left Leg) [mm]	Achilles Tendon Thickness (Right Leg) [mm]
**Induction**		
Group C	4.327 ± 0.502	4.313 ± 0.4389
Group ES	4.500 ± 0.5907	4.480 ± 0.3676
**Day 8**		
Group C	5.173 ± 1.529	4.693 ± 0.7488
Group ES	5.470 ± 1.105	5.170 ± 0.7439
**3 Months**		
Group C	5.100 ± 1.144	5.100 ± 1.313
Group ES	5.830 ± 1.643	5.310 ± 0.7340

**Table 4:** Achilles tendon thickness (left and right leg average) correlation with the VAS and VISA-A scores at all presentations for the control Group C (total number of patients N = 30), experimental Group ES (total number of patients N = 45), and all patients (total number of patients N = 75). Correlation (*p* < 0.05) at the level of significance α = 0.05 through the nonparametric Spearman test (does not assume Gaussian distribution).

	Nonparametric Spearman Correlation *p*-Value		
	Group C	Group ES	All Patients
VAS	<0.0001 (****)	0.0005 (***)	<0.0001 (****)
VISA-A	0.0118 (*)	<0.0001 (****)	<0.0001 (****)

## Data Availability

The original contributions presented in this study are included in the article. Further inquiries can be directed to the corresponding authors.

## Supplementary Materials

The following supporting information can be downloaded at: https://www.mdpi.com/article/10.3390/ph18040575/s1, Table S1: Anonymized patient data collected through the prospective randomized pilot study on the efficiency of a dietary supplementation regimen of vitamin E and selenium for the prevention of fluoroquinolone-induced tendinopathy; Table S2: CONSORT 2010 check-list.
