# Supplementary material for: A Prospective Randomized Pilot Study on the Efficacy of a Dietary Supplementation Regimen of Vitamin E and Selenium for the Prevention of Fluoroquinolone-Induced Tendinopathy
Source: Pharmaceuticals (Basel). 2025 Apr 15;18(4):575. doi: 10.3390/ph18040575 (PMC12030472; doi:10.3390/ph18040575)
Supplement: Supplementary file 1 [file pharmaceuticals-18-00575-s001.zip › pharmaceuticals-3574013-supplementary.pdf]

Table S1. Anonymized patient data collected through the prospective randomized pilot study on the efficiency of a dietary supplementation regimen of vitamin E and selenium for the prevention of fluoroquinolone-induced tendinopathy

| patient | age | left leg tendon Achilles thickness (measured in linear millimeters approximately 45 mm from the insertion of the tendon at the calcaneus level as a reference value) at the induction of treatment with levofloxacin 500 mg 1x1/day 7 days | right leg tendon Achilles thickness (measured in linear millimeters approximately 45 mm from the insertion of the tendon at the calcaneus level as a reference value) at the induction treatment with levofloxacin 500 mg 1x1/day 7 days | left leg tendon thickness (measured in linear millimeters approximately 45 mm from the insertion of the tendon at the calcaneus level as a reference value) achilles after 8 days from the start of treatment with levofloxacin 500 mg 1x1/day 7 days | right leg tendon Achilles thickness (measured in linear millimeters approximately 45 mm from the tendon insertion at the calcaneus level as a reference value) after 8 days from the start of treatment with levofloxacin 500 mg 1x1/day 7 days | left leg tendon Achilles thickness (measured in linear millimeters approximately 45 mm from the insertion of the tendon at the calcaneus level as a reference value) after 3 months from the start of treatment with levofloxacin 500 mg 1x1/day 7 days | right leg tendon Achilles thickness (measured in linear millimeters approximately 45 mm from the insertion of the tendon at the calcaneus level as a reference value) after 3 months after treatment with levofloxacin 500 mg 1x1/day 7 days | fibrillar disorganization of the Achilles tendon tenocytes | neovascularization documented sonographically | VAS score (from 0 painless to 10 maximum pain) of pain at the induction of treatment with levofloxacin 500 mg 1x1/day for 7 days |
|---|---|---|---|---|---|---|---|---|---|---|
| exp 1 | 58 | 4,8 | 4,5 | 8,9 | 6,2 | 6,6 | 9,1 | yes | yes | 0 |
| exp 2 | 57 | 3,8 | 5,3 | 4,2 | 3,6 | 5,5 | 5,9 | no | no | 0 |
| exp 3 | 60 | 3,9 | 4,4 | 4,3 | 4,6 | 4,3 | 4,5 | no | no | 0 |
| exp 4 | 40 | 3,5 | 3,7 | 3 | 3,5 | 3,5 | 3,7 | no | no | 0 |
| exp 5 | 60 | 4,6 | 4,1 | 5,2 | 4,5 | 5,2 | 4,9 | no | no | 0 |
| exp 6 | 43 | 4,7 | 4,2 | 5,1 | 4,7 | 5,2 | 4,5 | no | no | 0 |
| exp 7 | 59 | 4,2 | 3,9 | 4,7 | 4,4 | 4,4 | 5,7 | no | no | 0 |
| exp 8 | 32 | 3,8 | 3,9 | 4 | 4,3 | 3,9 | 4 | no | no | 0 |
| exp 9 | 43 | 4,1 | 4,2 | 5,7 | 4,7 | 5,8 | 4,5 | no | no | 0 |
| exp 10 | 50 | 5,2 | 4,5 | 5,5 | 4,9 | 5,4 | 4,8 | no | no | 0 |
| exp 11 | 32 | 3,9 | 4 | 4,4 | 4,3 | 4,1 | 4,3 | no | no | 0 |
| exp 12 | 44 | 4,5 | 4,1 | 4,7 | 4,3 | 4,6 | 4,2 | no | no | 0 |
| exp 13 | 34 | 4,5 | 4,2 | 4,5 | 5,6 | 4,5 | 5,3 | no | no | 0 |
| exp 14 | 43 | 5,1 | 4,6 | 5,2 | 4,9 | 5,5 | 4,9 | no | no | 0 |
| exp 15 | 53 | 4,3 | 5,1 | 8,2 | 5,9 | 8 | 6,2 | yes | yes | 0 |
| control 1 | 42 | 3,8 | 4,1 | 4,5 | 4,3 | 4,7 | 4,7 | no | no | 0 |
| control 2 | 36 | 4,2 | 4,5 | 7,3 | 5,1 | 8,5 | 5,3 | yes | yes | 0 |
| control 3 | 54 | 5,2 | 4,9 | 5,5 | 5,1 | 5,7 | 5,4 | no | no | 0 |
| control 4 | 33 | 4 | 4,7 | 7,2 | 5,6 | 9 | 6,1 | yes | yes | 0 |
| control 5 | 32 | 3,8 | 4,1 | 3,8 | 4,2 | 3,9 | 4,2 | no | no | 0 |
| control 6 | 41 | 5,1 | 4,7 | 5,2 | 4,9 | 5,2 | 4,8 | no | no | 0 |
| control 7 | 33 | 4,9 | 5 | 4,9 | 5,1 | 4,9 | 5,2 | no | no | 0 |
| control 8 | 60 | 5,3 | 4,6 | 5,5 | 4,9 | 5,3 | 4,9 | no | no | 0 |
| control 9 | 57 | 4,6 | 3,9 | 4,9 | 6,8 | 5,1 | 6,7 | yes | yes | 0 |
| control 10 | 60 | 4,1 | 4,3 | 5,9 | 5,7 | 6 | 5,8 | yes | yes | 0 |

| VISA A score (values from 100 completely asymptomatic to 0 maximum pain) specific for Achilles pain at the induction of treatment with levofloxacin 500 mg 1x1/day for 7 days | VAS (from 0 painless to 10 maximum pain) after 14 days of treatment with levofloxacin 500 mg 1x1/day 7 days | VISA A (values from 100 completely asymptomatic to 0 maximum pain) specific for achilles after 14 days from the induction of treatment with levofloxacin 500 mg 1x1/day for 7 days | VAS (from 0 to 10) after 3 months from the induction of treatment with levofloxacin 500 mg 1x1/day for 7 days | VISA A (values from 100 completely asymptomatic to 0 maximum pain) after 3 months from the induction of treatment with levofloxacin 500 mg 1x1/day for 7 days | serum selenium value at the induction of administration 200 micrograms/day for 28 days, normal serum values (23.00-190.00 micrograms/L) | serum selenium value after 28 days of administration of 200 micrograms/days. normal serum values (23.00-190.00 micrograms/L) | serum Vit E value at the induction of administration of 400 IU/day for 28 days. Normal serum values (5.00-20.00 mg/L) | serum Vit E value after 28 days of administration of 400 IU/day. Normal serum values (5.00-20.00mg/L) | patient |
|---|---|---|---|---|---|---|---|---|---|
| 90 | 5 | 48 | 3 | 68 | 102,8 | 117,4 | 19,58 | 26,11 | exp 1 |
| 94 | 0 | 85 | 0 | 90 | 94,7 | 102,9 | 18,24 | 25,12 | exp 2 |
| 81 | 0 | 71 | 0 | 81 | 81,1 | 118 | 16,77 | 24,86 | exp 3 |
| 94 | 0 | 85 | 0 | 90 | 109 | 118,4 | 18,22 | 27,8 | exp 4 |
| 94 | 0 | 85 | 0 | 94 | 101,3 | 110,5 | 12,46 | 22,21 | exp 5 |
| 90 | 0 | 83 | 0 | 90 | 96,72 | 105,8 | 16,82 | 25,37 | exp 6 |
| 85 | 1 | 67 | 0 | 75 | 101,2 | 119,4 | 13,22 | 22,19 | exp 7 |
| 94 | 0 | 75 | 0 | 90 | 85,3 | 124,6 | 10,43 | 18,54 | exp 8 |
| 87 | 0 | 73 | 0 | 84 | 73,4 | 110,3 | 12,35 | 19,58 | exp 9 |
| 90 | 1 | 68 | 0 | 75 | 89,82 | 103,7 | 7,32 | 18,6 | exp 10 |
| 87 | 0 | 75 | 0 | 81 | 104,4 | 125,7 | 9,54 | 18,77 | exp 11 |
| 83 | 0 | 83 | 0 | 90 | 93,8 | 125,8 | 15,69 | 24,95 | exp 12 |
| 81 | 0 | 75 | 0 | 85 | 103,9 | 116,2 | 17,41 | 26,93 | exp 13 |
| 90 | 0 | 90 | 0 | 90 | 98,5 | 120,1 | 11,6 | 23,67 | exp 14 |
| 85 | 6 | 48 | 3 | 67 | 102,5 | 119,1 | 18,9 | 28,3 | exp 15 |
| 100 | 0 | 94 | 0 | 94 | not tested | not tested | not tested | not tested | control 1 |
| 94 | 4 | 39 | 4 | 47 | not tested | not tested | not tested | not tested | control 2 |
| 85 | 1 | 75 | 1 | 75 | not tested | not tested | not tested | not tested | control 3 |
| 90 | 6 | 33 | 6 | 33 | not tested | not tested | not tested | not tested | control 4 |
| 83 | 1 | 64 | 1 | 64 | not tested | not tested | not tested | not tested | control 5 |
| 87 | 0 | 85 | 0 | 87 | not tested | not tested | not tested | not tested | control 6 |
| 85 | 0 | 90 | 0 | 84 | not tested | not tested | not tested | not tested | control 7 |
| 81 | 0 | 75 | 0 | 81 | not tested | not tested | not tested | not tested | control 8 |
| 94 | 5 | 44 | 4 | 56 | not tested | not tested | not tested | not tested | control 9 |
| 90 | 4 | 48 | 4 | 48 | not tested | not tested | not tested | not tested | control 10 |

# Table S2: CONSORT 2010 checklist of information to include when reporting a randomised trial*

| Section/Topic | Item No | Checklist item | Reported on page No |
|---|---|---|---|
| **Title and abstract** | | | |
| | 1a | Identification as a randomised trial in the title | 1 |
| | 1b | Structured summary of trial design, methods, results, and conclusions (for specific guidance see CONSORT for abstracts) | 1 |
| **Introduction** | | | |
| Background and objectives | 2a | Scientific background and explanation of rationale | 2-3 |
| | 2b | Specific objectives or hypotheses | 3 |
| **Methods** | | | |
| Trial design | 3a | Description of trial design (such as parallel, factorial) including allocation ratio | 10 |
| | 3b | Important changes to methods after trial commencement (such as eligibility criteria), with reasons | - |
| Participants | 4a | Eligibility criteria for participants | 10 |
| | 4b | Settings and locations where the data were collected | 10 |
| Interventions | 5 | The interventions for each group with sufficient details to allow replication, including how and when they were actually administered | 10-12 |
| Outcomes | 6a | Completely defined pre-specified primary and secondary outcome measures, including how and when they were assessed | 3,11 |
| | 6b | Any changes to trial outcomes after the trial commenced, with reasons | - |
| Sample size | 7a | How sample size was determined | - |
| | 7b | When applicable, explanation of any interim analyses and stopping guidelines | - |
| Randomisation: | | | |
| Sequence generation | 8a | Method used to generate the random allocation sequence | 11 |
| | 8b | Type of randomisation; details of any restriction (such as blocking and block size) | - |
| Allocation concealment mechanism | 9 | Mechanism used to implement the random allocation sequence (such as sequentially numbered containers), describing any steps taken to conceal the sequence until interventions were assigned | 11 |
| Implementation | 10 | Who generated the random allocation sequence, who enrolled participants, and who assigned participants to interventions | 11 |
| Blinding | 11a | If done, who was blinded after assignment to interventions (for example, participants, care providers, those | - |

| | | | |
|---|---|---|---|
| | | assessing outcomes) and how | |
| | 11b | If relevant, description of the similarity of interventions | - |
| Statistical methods | 12a | Statistical methods used to compare groups for primary and secondary outcomes | 11-12 |
| | 12b | Methods for additional analyses, such as subgroup analyses and adjusted analyses | - |
| **Results** | | | |
| Participant flow (a diagram is strongly recommended) | 13a | For each group, the numbers of participants who were randomly assigned, received intended treatment, and were analysed for the primary outcome | 10-11 |
| | 13b | For each group, losses and exclusions after randomisation, together with reasons | - |
| Recruitment | 14a | Dates defining the periods of recruitment and follow-up | 10-11 |
| | 14b | Why the trial ended or was stopped | - |
| Baseline data | 15 | A table showing baseline demographic and clinical characteristics for each group | 3 |
| Numbers analysed | 16 | For each group, number of participants (denominator) included in each analysis and whether the analysis was by original assigned groups | 4-8,10-12 |
| Outcomes and estimation | 17a | For each primary and secondary outcome, results for each group, and the estimated effect size and its precision (such as 95% confidence interval) | 4-8 |
| | 17b | For binary outcomes, presentation of both absolute and relative effect sizes is recommended | - |
| Ancillary analyses | 18 | Results of any other analyses performed, including subgroup analyses and adjusted analyses, distinguishing pre-specified from exploratory | - |
| Harms | 19 | All important harms or unintended effects in each group (for specific guidance see CONSORT for harms) | - |
| **Discussion** | | | |
| Limitations | 20 | Trial limitations, addressing sources of potential bias, imprecision, and, if relevant, multiplicity of analyses | 8-10 |
| Generalisability | 21 | Generalisability (external validity, applicability) of the trial findings | 8-10 |
| Interpretation | 22 | Interpretation consistent with results, balancing benefits and harms, and considering other relevant evidence | 8-9 |
| **Other information** | | | |
| Registration | 23 | Registration number and name of trial registry | - |
| Protocol | 24 | Where the full trial protocol can be accessed, if available | 10-12 |
| Funding | 25 | Sources of funding and other support (such as supply of drugs), role of funders | 12 |

*We strongly recommend reading this statement in conjunction with the CONSORT 2010 Explanation and Elaboration for important clarifications on all the items. If relevant, we also recommend reading CONSORT extensions for cluster randomised trials, non-inferiority and equivalence trials, non-pharmacological treatments, herbal interventions, and pragmatic trials. Additional extensions are forthcoming: for those and for up to date references relevant to this checklist, see www.consort-statement.org.
